# A novel SNP in *COMT *is associated with alcohol dependence but not opiate or nicotine dependence: a case control study

**DOI:** 10.1186/1744-9081-7-51

**Published:** 2011-12-31

**Authors:** Joanne Voisey, Christopher D Swagell, Ian P Hughes, Bruce R Lawford, Ross MD Young, C Phillip Morris

**Affiliations:** 1 Institute of Health and Biomedical Innovation, Queensland University of Technology, Brisbane, Queensland, Australia; 2 Division of Mental Health, Royal Brisbane and Women's Hospital, Brisbane, Queensland, Australia

## Abstract

**Background:**

It is well established that *COMT *is a strong candidate gene for substance use disorder and schizophrenia. Recently we identified two SNPs in *COMT *(rs4680 and rs165774) that are associated with schizophrenia in an Australian cohort. Individuals with schizophrenia were more than twice as likely to carry the GG genotype compared to the AA genotype for both the rs165774 and rs4680 SNPs. Association of both rs4680 and rs165774 with substance dependence, a common comorbidity of schizophrenia has not been investigated.

**Methods:**

To determine whether COMT is important in substance dependence, rs165774 and rs4680 were genotyped and haplotyped in patients with nicotine, alcohol and opiate dependence.

**Results:**

The rs165774 SNP was associated with alcohol dependence. However, it was not associated with nicotine or opiate dependence. Individuals with alcohol dependence were more than twice as likely to carry the GG or AG genotypes compared to the AA genotype, indicating a dominant mode of inheritance. The rs4680 SNP showed a weak association with alcohol dependence at the allele level that did not reach significance at the genotype level but it was not associated with nicotine or opiate dependence. Analysis of rs165774/rs4680 haplotypes also revealed association with alcohol dependence with the G/G haplotype being almost 1.5 times more common in alcohol-dependent cases.

**Conclusions:**

Our study provides further support for the importance of the *COMT *in alcohol dependence in addition to schizophrenia. It is possible that the rs165774 SNP, in combination with rs4680, results in a common molecular variant of *COMT *that contributes to schizophrenia and alcohol dependence susceptibility. This is potentially important for future studies of comorbidity. As our participant numbers are limited our observations should be viewed with caution until they are independently replicated.

## Background

Catechol-O-methyl transferase (COMT) is an enzyme involved in the degradation of dopamine [1] and is encoded by the *COMT *gene. This lies in a region that has been strongly implicated in schizophrenia [2-4]. The *COMT *gene is located on chromosome 22q11 and association studies have identified a number of polymorphisms that are associated with schizophrenia [5-11]. One of the more interesting polymorphisms is rs4680 that encodes either valine or methionine at amino acid position 158, with the substitution shown to alter COMT enzyme activity [12]. The only other non-synonymous polymorphism in *COMT *that has been found to be associated with schizophrenia is rs6267. This alanine/serine substitution also alters COMT enzyme activity but has only been identified in Asian populations [13,14].

Along with schizophrenia, studies have also identified *COMT *associations with a range of psychiatric conditions. A haplotype including the rs4680 polymorphism is associated with risk for several anxiety disorders and major depression [15] and the rs4680 polymorphism has been associated with panic disorder [16] and early onset major depression in a large European study [17]. Another European study found that *COMT *variation could predict onset of depressive episodes following exposure to stressful life events [18]. The rs4680 polymorphism has been studied in addictive behaviours and found to contribute significantly to the development of late-onset alcohol dependence [19]. A Finnish study of 896 males found rs4680 to not only contribute significantly to alcohol intake in alcoholics but also in the general male population [20]. Smoking cessation and association with the rs4680 polymorphism was found in a large study from the Netherlands [21]. As well as nicotine and alcohol dependence, opiate dependence has also been studied for the rs4680 polymorphism. An association with opiate dependence and rs4680 was observed in Hispanic women but did not hold up after correction for multiple testing [22]. However there have been conflicting studies for *COMT *including a study that did not observe association with alcohol dependence or nicotine dependence [23]. Genome-wide association studies of nicotine and alcohol dependence have not found association with *COMT *to date [24-27].

Previously we confirmed that rs4680 is associated with schizophrenia in an Australian population but we have identified stronger association with schizophrenia with a novel *COMT *SNP, rs165774 [28]. Since psychotic disorders are often comorbid with alcohol and other substance dependence [29,30], we genotyped the two *COMT *polymorphisms in alcohol, nicotine and opiate dependence.

## Methods

### Participants

#### Controls

The control group consisted of 250 unrelated Caucasians (102 female and 148 male) with a mean age of 36.8 years (s.d. ± 12.8 years). The control group consisted of volunteers from the general public, hospital nursing and medical staff, and university staff and students. Formal screening for psychological disorders was not undertaken in the control population. As such the controls represent an unselected control group and may include individuals with substance dependence. To minimise population stratification bias, both control and clinical subjects were recruited in the Brisbane region (a city of approximately 2 million inhabitants on the East Coast of Australia) and all were of British or European descent (ascertained by subject self-reporting).

#### Opiate Dependence

A total of 120 unrelated Caucasian participants (50 female and 70 male) diagnosed as opiate-dependent were recruited for this study. All subjects were assessed using a checklist of specific criteria by a consultant psychiatrist or physician experienced in drug and alcohol dependence and met DSM-IV criteria for opiate dependence. All were being assessed for naltrexone treatment as outpatients in a large public hospital detoxification unit in Brisbane, Australia. Participants had a mean age of 30.0 years of age (s.d. ± 7.9 years). Approximately half of the participants were being managed on methadone prior to detoxification (47.2%) while the other half were on heroin (52.8%). Those on methadone had a mean dose of 48.1 milligrams (s.d. 30.5), with a range between 10-165 milligrams. The mean age of onset of heroin use was 22.4 years of age (s.d. 5.14), with a range between 15-43 years. Mean number of participant-reported detoxifications prior to this occasion was 3.5 (s.d. 3.3), with a range between 0-16 detoxifications. Cannabis was the most common concurrently used illicit substance reported by participants prior to treatment (52.5% reported use), followed by amphetamines (14.9% reported use). Opiate-dependent subjects were excluded from the study if they had an organic brain syndrome, psychosis or any other condition that would affect their ability to provide informed consent.

#### Alcohol Dependence

A total of 231 unrelated Caucasian (74 female and 157 male) alcohol-dependent subjects were recruited from large public hospitals in Brisbane, Australia. All subjects were assessed using a checklist of specific criteria by a clinical psychologist experienced in drug and alcohol dependence and met DSM-IV criteria of alcohol dependence. Participants had a mean age of 40.74 years (s.d. ± 10.3 years). All were inpatients and represented a spectrum of severity with a significant proportion (*n *= 65) of these patients being diagnosed with two or more alcohol related medical conditions such as pancreatitis, cirrhosis, hepatitis or peripheral neuropathy. Alcohol-dependent patients were excluded from the study if they had an organic brain syndrome, psychosis, or any other condition that would affect their ability to provide informed consent.

#### Nicotine Dependence

A total of 147 (68 male, 79 female) unrelated Caucasians with a mean age of 43.3 (s.d. ± 11.1 years) were recruited for smoking reduction with a view to eventual cessation through hospital and media advertisements. All subjects were assessed using a checklist of specific criteria by a consultant psychiatrist or physician experienced in drug and alcohol dependence and met DSM-IV criteria of nicotine dependence. All subjects were outpatients. Participant inclusion criteria included 18 years of age or older and smoking for at least three years and being generally healthy despite currently smoking 15 cigarettes or more per day. All were motivated to reduce smoking and had the goal of eventual cessation. However, all participants had at least one serious but unsuccessful attempt at quitting in the previous 24 months. One hundred and thirty nine of the participants were administered the Fagerstrom test for Nicotine Dependence [31].

Ethics approval was obtained from all institutions involved and each participant from the control, opiate dependence, alcohol dependence and nicotine dependence groups gave written informed consent.

### Genotyping

Oragene kits were used to extract DNA from saliva samples. Samples were genotyped using a homogeneous MassEXTEND (hME) Sequenom assay [32] performed by the Australian Genome Research Facility. The hME assay is based on the annealing of an oligonucleotide primer (hME primer) adjacent to the SNP of interest. The addition of a DNA polymerase along with a mixture of terminator nucleotides allows extension of the hME primer through the polymorphic site and generates allele-specific extension products, each having a unique molecular mass. The resultant masses of the extension products are then analysed by matrix-assisted laser desorption/ionization time-of-flight mass spectrometry (MALDI-TOF MS) and a genotype is assigned in real time. The hME assay was performed in multiplex with up to 36 reactions in a single well.

Primers sequences for rs165774 are as follows: PCR Primer 1: ACGTTGGATGGCCCTACCTAGCCAGGCAT; PCR Primer 2: ACGTTGGATGTCCCAGAAACTGGACACTGC; Extension Primer: cgctCCTCGTGCTCCTAGTC. Primers sequences for rs4680 are as follows: PCR Primer 1: ACGTTGGATGTTTTCCAGGTCTGACAACGG; PCR Primer 2: ACGTTGGATGACCCAGCGGATGGTGGATTT; Extension Primer: tcacGCACACCTTGTCCTTCA.

The genotyping fail rate was 3.75% for rs165774 and 6.92% for rs4680 for all included patients. The genotyping of several other SNPs were independently verified by other methods such as real-time PCR with a concordance rate of 96.6% (the *COMT *SNPs were not independently verified). Genotypes were determined by investigators blinded for clinical diagnoses.

### Statistical Analysis

A Pearson's chi-squared test was performed to identify statistical associations between alleles and genotype and substance dependence status. Odds ratios (OR) were also calculated. Tests were performed on both genotype and allele data. Statistical tests were performed using the COMPARE2 program from the WinPepi suite of epidemiology programs [33] and SPSS version 18. Hardy-Weinberg equilibrium (HWE) was computed using Utility Programs for Analysis of Genetic Linkage [34]. The analysis of genotypes under a recessive model involved pooling the low-risk homozygotes and the heterozygotes and comparing frequencies with the high-risk homozygotes, i.e. OR > 1. Correction for multiple testing was conducted using the Benjamini-Hochberg method [35]. Linkage disequilibrium and haplotype estimations were calculated using JLIN version 1.6.0 [36].

## Results

Two *COMT *SNPs (rs165774 and rs4680) which we had previously found to be associated with schizophrenia were genotyped in 250 controls, 147 nicotine-dependent subjects, 120 opiate-dependent subjects and 231 alcohol-dependent subjects to investigate their association with substance dependence. In addition to our previously reported association with schizophrenia, rs165774 was found to be associated with alcohol dependence at the allele level after correction for multiple testing. There was no association with opiate or nicotine dependence (Table 1). For the two groups (nicotine and opiate dependence) that did not show association with rs165774, we did not have sufficient power (< 0.80) to detect association with our sample size using retrospective power calculations for a case-control study in syntax SPSS version 18 because the odds ratios were so low (nicotine OR = 1.24 and opiate OR = 1.28). In fact a sample size ranging from 2000-3000 cases and controls would be required to show any significance for rs165774 with opiate or nicotine dependence with odds ratios of this size.

**Table 1:** Allele Association of *COMT *SNP rs165774

Sample	Allele frequency		χ^2^	*p*-value*	Odds Ratio	95% CI
	G	A				
Controls	304	176				
Nicotine dependence	199	93	1.856	0.173	1.24	0.91-1.69
Opiate dependence	161	73	2.072	0.150	1.28	0.91-1.78
Alcohol dependence	319	133	5.509	0.019	1.39	1.05-1.83

* *p*-value determined by Pearson's chi-squared test (DF = 1)

At the genotype level, rs165774 was still associated with alcohol dependence (Table 2). Individuals with alcohol dependence were more than twice as likely to carry the GG or AG genotype compared to the AA genotype. The rs165774 SNP was found to follow a dominant mode of inheritance for alcohol dependence when genotypes were pooled (*p *= 0.006, OR 2.24, 95% CI 1.2 to 4.3).

**Table 2:** Genotype Association of *COMT *SNP rs165774

Sample	Genotype Counts			*p*-value*
	AA (%)	AG (%)	GG (%)	
control	39 (16.25)	98 (40.83)	103 (42.92)	

Nicotine dependence	21 (14.38)	51 (34.93)	74 (50.69)	0.330
Odds ratio	1	0.966	1.334	0.205^†^
*p*-value		1.000	0.700	

Opiate dependence	13 (11.11)	47 (40.17)	57 (48.72)	0.363
Odds ratio	1	1.439	1.660	0.172^†^
*p*-value		0.626	0.300	
Alcohol dependence	18 (7.96)	97 (42.92)	111(49.12)	0.022
Odds ratio	1	2.146	2.336	0.023^†^
*p*-value		0.029	0.012	

* *p*-value determined by Pearson's χ^2 ^test (DF = 2)

^† ^*p*-value determined using the extended Mantel-Haenszel test for trend (DF = 1)

In addition to our previously reported association with schizophrenia, rs4680 was only marginally associated with alcohol dependence at the allele level (Table 3) but was not significant with opiate or nicotine dependence. However the association with rs4680 and alcohol dependence did not hold up at the genotype level (*p *= 0.135) and it did not survive correction for multiple testing. For the two groups (nicotine and opiate dependence) that did not show association with rs4680, the odds ratios were even lower (nicotine OR = 1.03 and opiate OR = 1.24) requiring a sample size ranging from 2000-200000 cases and controls.

**Table 3:** Allele Association of *COMT *SNP rs4680

Sample	Allele frequency		χ^2^	*p*-value*	Odds Ratio	95% CI
	G	A				
Controls	194	244				
Nicotine dependence	126	154	0.035	0.852	1.03	0.76-1.39
Opiate dependence	110	112	1.639	0.200	1.24	0.89-1.71
Alcohol dependence	231	223	3.880	0.049	1.30	1.00-1.69

* *p*-value determined by Pearson's chi-squared test (DF = 1)

Analysis of combined addictive disorders (alcohol, nicotine and opiate dependence) revealed no association at the genotype level (*p *= 0.18) or allele level for rs4680 (*p *= 0.114). Combined addictive disorders were associated with rs165774 at the allele level (*p *= 0.020) but not at the genotype level (*p *= 0.062).

In addition, the nicotine-dependent group genotype was tested for an association with the Fagerstrom Nicotine Dependence Test (FNDT). Analysis of variance revealed no significant associations between FNDT and genotype (rs165774: *p = *0.135, *F = *2.032; rs4680: *p *= 0.520, *F *= 0.656).

Linkage disequilibrium (LD) analysis was performed on all groups for the rs4680 and rs165774 SNPs. Strong LD was found between the two SNPs in all groups. Controls: D' = 0.93, r^2 ^= 0.37. Nicotine: D' = 1.0, r^2 ^= 0.37. Opiate: D' = 0.90, r^2 ^= 0.35. Alcohol: D' = 0.98, r^2 ^= 0.41. Haplotype analysis of rs165774 and rs4680 was performed in all groups and only reached statistical significance in the alcohol-dependent group (Table 4). When all substance dependence groups were analysed together as a single phenotype, the haplotypes did not show association.

**Table 4:** Haplotype analysis of *COMT *rs4680 and rs165774 polymorphisms in patients with alcohol dependence

Haplotype rs4680/rs165774	Controls^c^ (%)	Alcohol^c^ (%)	Odds Ratio^a ^(*P*)	χ^2 b^	*P*
A/A	147 (34.5)	126 (28.4)	1	3.794	0.051
A/G	93 (21.8)	89 (20.0)	1.116 (1.000)	0.419	0.517
G/A	5 (1.2)	1 (0.2)	0.233 (0.392)	3099	0.078
G/G	181 (42.5)	228 (51.4)	1.470 (0.042)	6.866	0.009

TOTAL	426	444		9.656	0.022

^a ^Odds ratio with respect to rs4680 A/rs165774 A haplotype which is more commonly associated with a normal phenotype

^b ^Likelihood ratio χ^2 ^test comparing haplotype frequency between groups when all other haplotypes were pooled (DF = 1, and DF = 3 for total χ^2^)

^c ^Haplotypes frequencies were estimated using JLIN version 1.6.0

Respective genotype frequencies indicated that both polymorphisms were in HWE in both case and control samples, with the exception of rs165774 in the nicotine group (χ^2 ^= 5.6, *p = *0.02), although this SNP did not show association with the nicotine dependence group.

## Discussion

Two SNPs in *COMT *previously identified as being associated with schizophrenia [28] were examined for their association with alcohol, nicotine and opiate dependence. Evidence suggests that a common molecular axis involving the dopamine pathway exists for multiple psychiatric disorders. One SNP in *COMT *rs4680 (Val 158 Met) has received considerable scientific attention as it influences the availability of COMT and subsequently the efficiency of extracellular dopamine degradation. Homozygosity for 158 Met leads to a 3-4 fold reduction in enzymatic activity compared with homozygosity for 158 Val [37,38]. Since COMT plays a crucial role in the metabolism of dopamine it is likely that it contributes to the etiology of alcohol dependence. Alcohol-induced euphoria is associated with rapid release of dopamine in limbic areas and a study has shown that subjects with the 158 Met polymorphism have low dopamine inactivation and are more vulnerable to alcohol dependence [19]. It is well known that psychotic disorders are frequently comorbid with alcohol and other substance dependence [29,30] but it is not possible to tell whether this is because these disorders have a common molecular metabolic aetiology or whether psychosis leads to substance abuse via an as yet unidentified mechanism.

Association studies of rs4680 have produced varied results and it may play a role in susceptibility to a range of psychiatric conditions. Another possible candidate regarding risk is rs165774 that we have previously shown to be associated with schizophrenia and is in strong linkage disequilibrium with rs4680 [28]. Most studies of *COMT *have focused on rs4680 and a stronger candidate polymorphism is yet to be identified. Analysis of rs165774 revealed associations with alcohol dependence at both the allele and genotype levels. Although rs4680 was associated with alcohol dependence at the allele level, the association was not convincing and did not hold up at the genotype level. Nicotine and opiate dependence were not associated with rs4680 or rs165774 in our study but association cannot be ruled out as we only analysed a limited number of cases (Tables 1 and 3).

The only other study besides our schizophrenia study [28] that has found association with a neurological/psychiatric condition and rs165774 is a Chinese study examining mental retardation in a Chinese Han population [39]. The rs165774 SNP has not been studied in genome-wide association studies.

## Conclusions

This study identified a polymorphism in *COMT *that possibly plays a role in psychiatric liability for schizophrenia and alcohol dependence. The rs165774 polymorphism may affect *COMT *enzyme activity and therefore dopamine inactivation resulting in vulnerability to substance dependence. The rs165774 SNP displayed stronger association with alcohol dependence than rs4680 but it is in tight LD with the non-synonymous SNP rs4680. A limitation of our study is that controls were not screened for psychiatric conditions including substance dependence. However not screening controls is unlikely to bias results as screening would be likely to strengthen the associations observed between cases and controls. Another limitation was that no SCID (Structured Clinical Interview for DSM Disorders) was performed on patients. However, all participants met DSM-IV criteria. We did not adjust our P-values for multiple testing as the validity of this technique is controversial and has been shown to increase type II errors, making important differences non-significant [40]. Therefore, our significant association must be seen as exploratory and further replication is required. For the opiate and nicotine-dependent groups that did not show significance with rs165774 or rs4680, replication in an independent study is required with a larger sample size.

## Competing interests

All authors declare that they have no conflicts of interest, including no financial, personal or other relationships with other people or organisations.

## Authors' contributions

JV: Involved in conception and design, acquisition of data, analysed and interpreted data, drafted the article and approved final version. CS: Involved in conception and design, acquisition of data, critically revised article and approved final version. IH: Involved in conception and design, acquisition of data, analysed and interpreted data, critically revised article and approved final version. BL: Involved in conception and design, acquisition of data, critically revised article and approved final version. RY: Involved in conception and design, acquisition of data, critically revised article and approved final version. CM: Involved in conception and design, acquisition of data, critically revised article and approved final version.
